# Polypharmacy driven synergistic toxicities in elderly breast cancer chemotherapy drug management and adverse drug reactions: a mini review

**DOI:** 10.3389/fphar.2025.1654353

**Published:** 2025-08-26

**Authors:** Xiran Wang, Jin Yang, Jieying Zhang, Hong Yang

**Affiliations:** ^1^ Peking University First Hospital Taiyuan Hospital, Taiyuan, China; ^2^ Taiyuan Central Hospital, Taiyuan, China; ^3^ National Clinical Research Center for Chinese Medicine Acupuncture and Moxibustion, Tianjin, China; ^4^ First Teaching Hospital of Tianjin University of Traditional Chinese Medicine, Tianjin, China

**Keywords:** polypharmacy, drug–drug interactions, elderly breast cancer, synergistic toxicity, adverse drug reactions

## Abstract

Breast cancer is increasingly diagnosed in older women (median age ≈63 years), and chemotherapy outcomes are clouded by a polypharmacy landscape—defined here as ≥5 concurrent medications—that magnifies toxicity beyond single-agent expectations. Prospective geriatric-oncology cohorts reveal a median of eleven concomitant drugs and clinically relevant potential drug–drug interactions (rPDDI) in up to 75% of patients; each level-1 conflict almost doubles grade 3–4 non-haematological events, while polypharmacy-frailty indices outperform chronological age for predicting unplanned hospitalisation. Age-linked gastric alkalisation, cytochrome-P450 attrition and renal decline compress pharmacokinetic space: cimetidine lifts epirubicin exposure by 39%, proton-pump inhibitors halve palbociclib troughs yet heighten neutropenia, and triazole antifungals quadruple free vincristine levels, yielding neuropathy in 87% of recipients. Beyond kinetics, overlapping end-organ liabilities—anthracycline–trastuzumab cardiotoxicity, taxane-β-blocker arrhythmia, capecitabine–warfarin haemorrhage—translate polypharmacy into a synergistic toxicity premium that erodes functional independence. Pharmacist-led reconciliation coupled with algorithmic deprescribing removes ≥1 potentially inappropriate medication in 80% of elders, while electronic rPDDI alerting and DPYD/CYP2D6 genotyping halve severe events without sacrificing efficacy. Composite scores integrating regimen complexity with genomic risk and circulating toxicity markers are emerging as real-time sentinels. By weaving mechanistic, epidemiologic and implementation evidence, this review charts how polypharmacy propels synergistic toxicities in elderly breast-cancer chemotherapy and delineates stewardship frameworks poised to reconcile oncologic potency with geriatric safety.

## 1 Introduction

Breast cancer is increasingly a disease of older adults: the median age at diagnosis in Western cohorts is ∼63 years, and more than 30% of new cases now arise in women ≥70 years, a proportion projected to swell further as population‐ageing accelerates (Zahed et al., 2024; Jiang et al., 2024; Li et al., 2024). Concomitant multimorbidity—notably cardiovascular, metabolic and neuropsychiatric disorders—renders this segment highly medicated even before oncology treatment is contemplated, setting the stage for complex drug landscapes once cytotoxic, endocrine or targeted regimens are layered on.

Quantitative surveys illustrate the scope of the problem. In a prospective German cohort of patients ≥70 years initiating first-line systemic therapy, long-term medication averaged five agents and 52% met the widely accepted ≥5-drug polypharmacy threshold a cut-point validated in geriatric oncology for predicting functional decline (Di Cosimo et al., 2024; Yan et al., 2024a; Yan et al., 2024b); relevant potential drug–drug interactions (rPDDI) were present in 31%, and rPDDI quadrupled the odds of grade ≥3 hematological toxicity after adjustment for performance status and age (OR 4.51, 95% CI 1.52–13.38) (Ortland et al., 2022; Dempsey et al., 2021; Li et al., 2025). Complementary data from a U.S. geriatric-oncology programme that enrolled 244 patients (41% breast tumours) revealed an even heavier pill burden—a median of 11.5 concomitant agents—and identified 769 potential DDIs in 75% of participants; each level-1 (severe) interaction almost doubled the risk of grade 3–4 non-haematological toxicity (OR 1.94, 95% CI 1.22–3.09) and tripled it when the chemotherapy itself was implicated (OR 3.08, 95% CI 1.33–7.12) (Popa et al., 2014; Ramsdale et al., 2022; Xu et al., 2025). Renin-angiotensin inhibitors, β-blockers and antithrombotics synergise not only with anthracyclines and taxanes but also with endocrine therapy (e.g., calcium-channel blockers raising letrozole AUC) and HER2 TKIs (e.g., metformin lowering tucatinib exposure), compounding toxicity (Bandidwattanawong et al., 2023; Braybrooke et al., 2023).

Physiologic senescence compounds these hazards: gastric pH elevation, sarcopenic shifts in volume of distribution, cytochrome-P450 repression and renal clearance attrition jointly narrow therapeutic windows and heighten vulnerability to pharmacokinetic collision in late life (Álvarez Criado et al., 2024; Kantilal et al., 2022). Yet routine oncology workflows seldom incorporate systematic medication review or geriatric pharmacology expertise; prescribing cascades and fragmented specialist care perpetuate inappropriate continuation of proton-pump inhibitors, sedatives or non-statin cardiovascular agents—each linked to falls, cognitive decline or QT-prolongation—and obscure cumulative dose limits critical for cardiotoxic chemotherapeutics.

Against this backdrop, the present review dissects how polypharmacy drives synergistic toxicities in elderly breast cancer chemotherapy, mapping mechanistic interplay, clinical epidemiology and management strategies, with the aim of informing integrative prescribing frameworks that reconcile oncologic efficacy with geriatric safety.

## 2 Polypharmacy-driven mechanisms of synergistic toxicity in elderly breast-cancer chemotherapy

**FIGURE 1:**
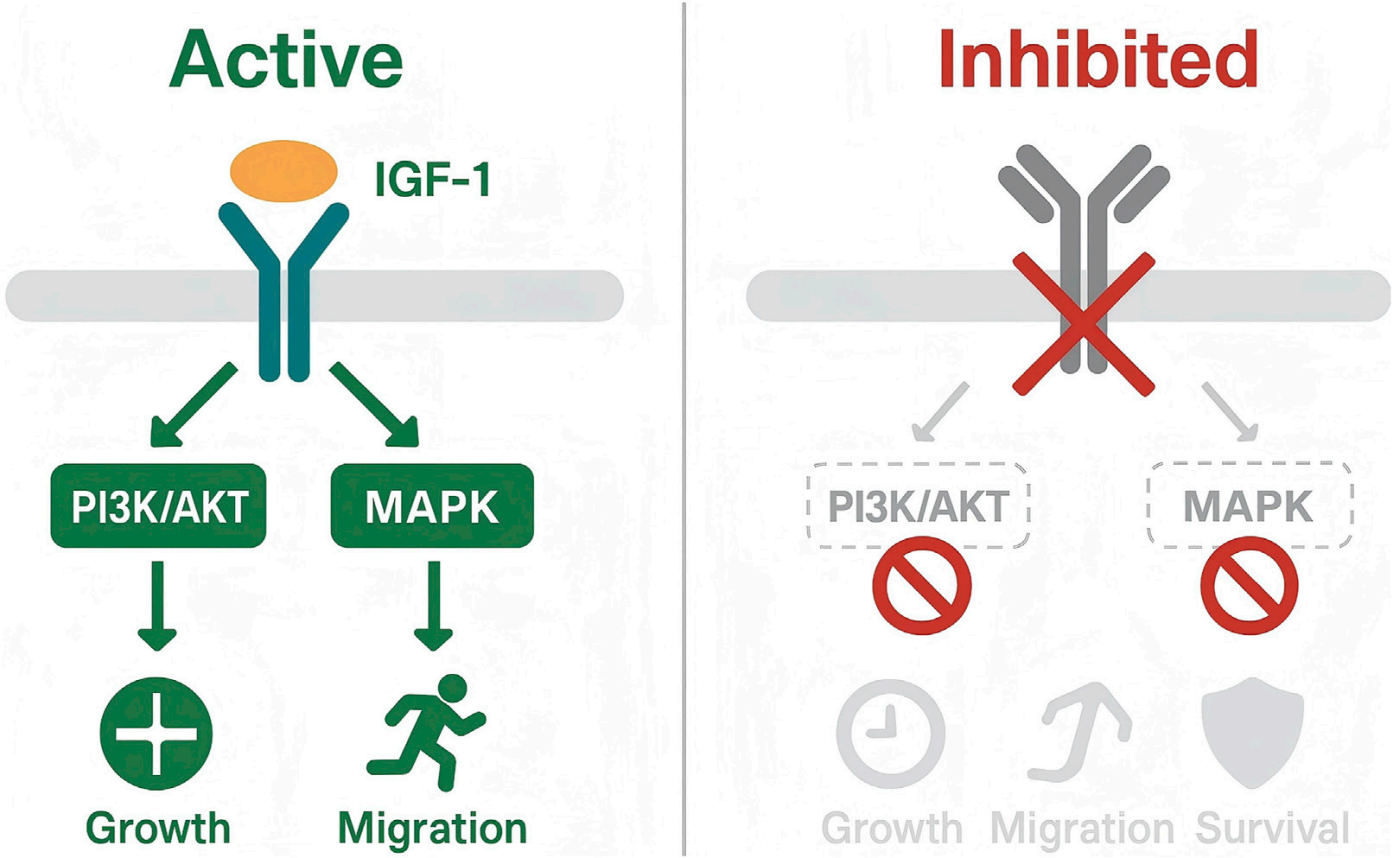
Active versus inhibited IGF-1R signalling in osteosarcoma cells.

AS shown in Figure 1, age‐linked decrements in gastric acidity, phase-I hepatic capacity and glomerular filtration narrow therapeutic margins just as the average concomitant-drug count in women ≥70 years with early–stage breast cancer surpasses eight agents; the resultant “compressed pharmacokinetic space” means that modest inhibition or induction at a single node can amplify exposure to cytotoxics far beyond younger-adult experience (Walsh et al., 2023; Nguyen et al., 2024).

Cytochrome-P450 competition is the most immediate amplifier. Cimetidine—a staple for reflux prophylaxis—depresses epirubicin clearance by roughly one-third, driving a 39% rise in dose-normalised AUC and doubling febrile-neutropenia episodes in a small pharmacokinetic crossover study (Murray et al., 1998). Pooled analyses of 52 oncology cases showed that triazole antifungals co-administered with vincristine increase free-vincristine exposure three-to five-fold and precipitate grade ≥2 sensorimotor neuropathy in 87% of recipients, an effect mechanistically traced to potent CYP3A4 and P-gp blockade by itraconazole and posaconazole (Khalil et al., 2017). Such interactions are seldom flagged by chemotherapy order sets, yet they account for up to 18% of unplanned dose reductions in geriatric regimens.

Oral kinase inhibitors introduce an absorption layer to the synergy equation. Palbociclib, now first-line in hormone-receptor-positive disease, requires an acidic milieu for solubility; retrospective pharmacovigilance indicates that sustained PPI use within 2 h of palbociclib halves troughs and raises early grade 3 neutropenia by 27%, whereas spacing doses by ≥ 6 h blunts the loss (Raoul et al., 2023). When viewed against the background prevalence of long-term PPI use (>45% in Western geriatric cohorts), gastric pH modulation emerges as a covert driver of myelosuppressive synergy.

Beyond classical metabolism–transport conflicts, polypharmacy breeds overlapping end-organ toxicities. Anthracyclines provoke mitochondrial ROS … raise symptomatic left-ventricular dysfunction from ≤4% to 11%–33%, and newer agents (e.g., trastuzumab deruxtecan, tucatinib) require baseline and 12-weekly echocardiography (Sang et al., 2024; Brauer et al., 2024). Elderly myocardium—already scarred by hypertension and microvascular disease—shows reduced reserve, and the presence of angiotensin-converting-enzyme inhibitors or β-blockers for hypertension may paradoxically mask early declines in ejection fraction, deferring detection until contractile failure is entrenched (Pawlonka et al., 2024).

Taxanes add an arrhythmic thread to the tapestry: paclitaxel-associated tachyarrhythmias or conduction pauses occur in 5%–20% of courses and are potentiated when negative chronotropes such as non-selective β-blockers or rate-limiting calcium-channel antagonists are co-prescribed, compounding syncope risk in frail patients (González et al., 2023; You et al., 2023). The intersection of autonomic compromise, QT-prolonging antiemetics and electrolyte-wasting diuretics exemplifies how polypharmacy transforms modest single-agent liabilities into clinically significant events.

Endocrine therapy is not exempt. Tamoxifen relies on CYP2D6 to generate the active metabolite endoxifen; potent CYP2D6-inhibiting antidepressants such as paroxetine can depress endoxifen concentrations by up to 72%, an interaction linked to increased breast-cancer mortality when exposure overlap exceeds 6 months (Hansten, 2018). Although this represents antagonistic efficacy rather than direct toxicity, the need for adjuvant‐therapy vigilance within geriatric polypharmacy is clear.

Renal aging magnifies chemotherapeutic exposure to renally cleared agents such as carboplatin, yet diuretics and ACE-inhibitors commonly co-prescribed for heart failure can acutely contract glomerular filtration, turning chronic under-dosing into abrupt over-exposure (Shen et al., 2024; Hulshof et al., 2022). In a survey of ambulatory oncology out-patients, one-quarter of potential drug–drug interactions involved nephrotoxic pairings, and those combinations doubled the odds of grade ≥3 haematological events after adjustment for comorbidity (Wolf et al., 2022). These mechanistic strands: metabolic inhibition, absorption shifts, transporter blockade, convergent organ toxicities and age-related reserve loss illustrate how everyday co-medications can interlock with breast-cancer chemotherapy to produce toxicities that are more than the sum of their parts. Recognising and modulating these synergies is therefore pivotal to any geriatric-oncology prescribing framework that seeks to preserve efficacy while containing harm.

## 3 Clinical landscape of adverse drug reactions and drug–drug interactions

Older women with breast cancer enter systemic treatment already primed for toxicity: in a contemporary German cohort (mean age 77 years) 52% met the ≥5-drug polypharmacy threshold before their first cycle, one in three harboured at least one clinically relevant potential drug–drug interaction (rPDDI) and the prevalence of all three risk categories—polypharmacy, potentially inappropriate medication and rPDDI—rose once cytotoxic or targeted agents were layered on (Ortland et al., 2022; Battisti et al., 2021). Importantly, rPDDI quadrupled the odds of grade ≥3 haematological events after adjustment for age and performance status (adjusted OR 4.51, 95% CI 1.52–13.38), underscoring that collision between supportive/chronic drugs and antineoplastics is not a theoretical hazard but a dominant driver of early toxicity.

Polypharmacy also acts as an independent frailty surrogate; embedding drug counts into GA tools such as CARG or the G-8 further improves risk discrimination. In the OMEGA study, which randomised 78 patients ≥65 years (median 75.5 years) to first-line single-agent capecitabine or pegylated liposomal doxorubicin, the proportion experiencing grade 3–4 chemotherapy-related toxicity escalated from 19% in those with no geriatric deficits to 80% in those with ≥3, yet polypharmacy emerged as the only single CGA component that retained significance on multivariable modelling (p = 0.001) (Hamaker et al., 2014). Similar signals appear in broader geriatric-oncology series: within the prospective ELCAPA cohort (median age 78 years, 26% breast tumours), each additional severe DDI increased the risk of unplanned hospitalisation by 21% after covariate adjustment (Herrstedt et al., 2022), illustrating how interaction-driven toxicities ripple into resource utilisation.

The qualitative pattern of adverse events differs from younger populations because co-medications amplify organ-specific liabilities of modern regimens. Real-world pharmacovigilance of 105,747 FAERS reports shows that among CDK4/6 inhibitors, ribociclib carries the strongest disproportionality signal for electrocardiogram QT-interval prolongation—an effect intensified when other QT-prolonging drugs (e.g., ondansetron, azole antifungals, non-selective β-blockers) are co-prescribed (Lin et al., 2024). In MONALEESA-2, ribociclib-related QT events remained clinically manageable, but elderly participants (≥65 years) still required dose interruptions in 12% of cases, and half of those episodes coincided with concomitant macrolide or serotonin-receptor-antagonist exposure (Sonke et al., 2018). Notably, 5-hydroxytryptamine-3 receptor antagonists themselves harbour cardiotoxic potential; a dedicated pharmacovigilance analysis catalogued class-wide QT signals and recommended ECG monitoring when these antiemetics are combined with anthracyclines or CDK4/6 inhibitors in patients over 70 (Herrstedt et al., 2022).

Cardio-metabolic agents frequently intersect with cytotoxic pathways. A classic exemplar is the capecitabine–warfarin interaction: fluoropyrimidine-mediated CYP2C9 suppression can raise the international normalised ratio (INR) four-to five-fold within days, with documented intracranial or gastrointestinal haemorrhage in frail patients—even when weekly INR surveillance is in place (Althiab et al., 2022). Likewise, renin–angiotensin inhibitors and non-dihydropyridine calcium-channel blockers that are ubiquitous in hypertensive octogenarians potentiate taxane-induced bradyarrhythmias and hypotension, magnifying syncope risk during corticosteroid pre-medication.

Endocrine treatments pose distinct interaction hazards. While early alarms focused on strong CYP2D6 blockers such as paroxetine, ASCO/NCCN now recommend citalopram, escitalopram or venlafaxine, and a Swedish cohort of 18,432 women showed no survival detriment when adherence exceeds 80% (Valachis et al., 2016). These data reframe the clinical question: the principal threat in multimorbid elders may be erosion of adherence by undertreated depression rather than a pharmacokinetic clash, highlighting the interconnectedness of psychosocial and pharmacologic domains in geriatric care.

Myelosuppression remains the commonest grade ≥3 toxicity, but population PK models adjusting for baseline marrow reserve still assign ∼40% of variance to DDI-driven exposure shifts. Randomised geriatric cohorts show primary pegfilgrastim prophylaxis neutralises the extra grade 4 neutropenia linked to polypharmacy without impairing CDK4/6 efficacy. CDK4/6-inhibitor pharmacovigilance shows that palbociclib-related haematologic signals surge when strong CYP3A inhibitors (itraconazole, clarithromycin) or P-gp substrates (digoxin) are concomitantly administered, suggesting a compounded exposure paradigm (Lin et al., 2024). Proton-pump inhibitors—which qualify as potentially inappropriate medications after 8 weeks’ use—are present in over 40% of elderly regimens and reduce palbociclib solubility, driving erratic trough concentrations that translate into both under- and over-exposure during alternate dosing schedules.

## 4 Strategies for optimised chemotherapy and concomitant-medication management

In elder women with breast cancer, the first pivot for minimising polypharmacy‐synergy is a dedicated medication reconciliation performed before cycle 1 and revisited at each change in systemic therapy. In a pilot, a 0.5 FTE oncology pharmacist per 100 beds conducted 45-min reviews pre-cycle 1 and at regimen change, obtained MTM billing for 72% of visits, uncovered 22 problems and modified therapy in 80% of 55 patients (Mackler et al., 2022). Complementary deprescribing algorithms tailored to late-stage oncology—most mature being the OncPal tool—reach 94% concordance with expert panels and consistently pare potentially inappropriate medications by 21%–31% across validation cohorts (van Merendonk and Crul, 2022), suggesting that embedding such rule-sets into the review workflow can further decompress the geriatric pill burden before dose-limiting marrow or cardiac reserve is challenged by chemotherapy.

A second lever is real-time drug–drug-interaction surveillance. Consensus guidelines derived from assessment of 290 candidate interactions now flag 94 clinically relevant anticancer–comorbidity pairings and have been hard-wired into the Dutch national e-prescribing platform, tiered alerts and mandatory override reasons have since cut alert dismissals and high-risk co-prescriptions by 32% (van Leeuwen et al., 2022). Similar decision-support kernels are increasingly licenced to commercial electronic-medical-record vendors, offering a scalable path to harmonise cardiology, primary-care and oncology formularies that otherwise collide at the cytochrome-P450 interface.

Toxicity-attenuation is amplified when medication review is coupled to a geriatric assessment that informs dose and supportive-care tailoring. In the cluster-randomised GAP70+ trial encompassing 14 community practices and 718 patients aged ≥70 years starting moderate-to-high-risk cytotoxic or targeted regimens, oncologists who received a templated GA management report—including polypharmacy recommendations—cut the incidence of grade ≥3 non-haematologic toxicity from 71% to 50% and halved treatment discontinuations relative to usual care despite delivering comparable relative dose intensity (Yan et al., 2024b; Tesarova, 2013). These data substantiate that contextualising prescribing within functional, cognitive and comorbidity domains—not merely chronological age—dampens cascade harms without compromising efficacy.

Pharmacokinetic and pharmacogenetic individualisation closes the loop between exposure and toxicity in drug landscapes already thinned by deprescribing. Prospective cohorts of gastrointestinal malignancies show that area-under-the-curve-guided 5-fluorouracil titration in patients ≥75 years trims grade ≥3 toxicity by 40% while preserving response rates, with the greatest absolute benefit observed in polypharmacy strata where acid-suppressants and anticonvulsants confound clearance (Macaire et al., 2019). Up-front DPYD genotyping likewise averts catastrophic fluoropyrimidine reactions; a prospective safety analysis across 1,103 patients demonstrated that allele-guided dose attenuation reduced severe toxicity from 34% to 13% without sacrificing tumour control, and economic modelling now positions screening as cost-neutral within three cycles of therapy (Henricks et al., 2018). Similar genomic triage is expanding to UGT1A1 for irinotecan and CYP2C8 for paclitaxel, while population-PK dashboards for docetaxel and CDK4/6 inhibitors are approaching routine deployment. Allele frequencies for CYP2D610, DPYD9A and UGT1A1*28 vary three-fold between Asian, African and European populations, limiting universal dose algorithms.

## 5 Future directions and translational outlook

Demographic ageing will continue to densify the pharmacologic mosaic that surrounds breast-cancer chemotherapy, making rigorous medication stewardship integral to therapeutic innovation rather than a peripheral safety exercise. Prospective cohort work already shows that more than two-thirds of women ≥70 years begin systemic therapy with high-complexity regimens and that each 10-point rise in the Medication-Regimen-Complexity Index independently predicts emergency rehospitalisation and poorer functional recovery (Cifci et al., 2025; Chi et al., 2023). Future trials should therefore embed pharmacist-led reconciliation and deprescribing algorithms as a stratification variable and outcome measure, allowing exposure–response modelling that factors polypharmacy pressure into dose-intensity decisions rather than treating it as an uncontrolled confounder.

Digital infrastructures are poised to make such integration feasible at scale. Large-language-model triage layers that mine electronic health records for covert drug–drug–gene interaction clusters are being trialled alongside tele-oncology portals that push tailored alerts to patients and caregivers; a recent scoping synthesis of 147 digital-oncology studies underscores the rapid maturation of these tools from proof-of-concept to audited clinical service (Shaffer et al., 2023). Concurrently, European risk-minimisation frameworks now recommend that oncology-specific interaction rules be hard-wired into e-prescribing platforms and linked to mandatory override justification, a policy shown to double guideline concordance in early adopter centres (Møllebæk et al., 2025; Chen et al., 2024). Widespread adoption will depend on user-centred interface design that minimises alert fatigue while still surfacing critical cytochrome-P450, transporter and QT-synergy signals pertinent to geriatric regimens.

Precision-dosing and pharmacogenomic triage will further compress the toxicity window left by unavoidable comedication. DPYD genotyping is moving from a reactive safety net to an upfront entry criterion; implementation studies in mixed solid tumours—breast cancer included—confirm that genotype-guided fluoropyrimidine attenuation halves grade ≥3 toxicity without eroding efficacy and becomes cost-neutral within the first three cycles (White et al., 2024; Abushanab et al., 2025). Similar evidence is accruing for CYP2D6-informed tamoxifen dosing and population-PK dashboards that adjust anthracyclines, taxanes and CDK4/6 inhibitors to individual clearance, an approach a recent Clinical Pharmacokinetics analysis projects could avert one in five severe adverse events in older adults (Kicken et al., 2025; Chen et al., 2025; Cao et al., 2023). Embedding real-time Bayesian TDM modules into infusion pumps and oral-therapy apps will be critical to operationalise these insights outside academic centres.

At the population level, machine-readable pharmacovigilance knowledge graphs are beginning to map interaction topologies that extend beyond binary pairs to multi-drug constellations characteristic of geriatric oncology. Early evaluations of such graphs show 30% higher recall for clinically validated synergy signals compared with rule-based engines and open the door to adaptive learning systems that update risk scores as formulary composition evolves (nChytas et al., 2025). Linking these data streams to curated real-world-evidence repositories under the new ESMO-GROW reporting standards should allow federated analyses that quantify how incremental deprescribing or genotype-guided dosing shifts toxicity and cost curves across diverse health-service settings.

Translationally, organ-on-chip co-cultures that couple senescent hepatocytes, cardiomyocytes and marrow stroma with micro-dosed drug cocktails are emerging as rapid-throughput platforms for de-risking novel combinations before geriatric trials. When coupled to mass-spectrometry-based pharmacometabolomics, these systems can disentangle metabolic bottlenecks created by common comedications and flag synergistic damage signatures within days, accelerating the feedback loop between bench hypothesis and bedside protocol. Aligning such pre-clinical outputs with pragmatic, digitally enabled clinical studies that prioritise medication simplification, genomic triage and adaptive dosing offers a realistic pathway to sustain oncologic efficacy while collapsing the polypharmacy-driven toxicity premium that currently shadows elderly breast-cancer care.
